# Immunomodulatory peptide–drug conjugate MEL-dKLA suppresses progression of prostate cancer by eliminating M2-like tumor-associated macrophages

**DOI:** 10.3389/fimmu.2025.1652166

**Published:** 2025-09-12

**Authors:** Ik-Hwan Han, Ilseob Choi, Soyoung Kim, Minjin Kwon, Hyojung Choi, Hyunsu Bae

**Affiliations:** Department of Physiology, College of Korean Medicine, Kyung Hee University, Seoul, Republic of Korea

**Keywords:** prostate cancer, peptide drug conjugates, tumor associated-macrophages, tumor microenvironment, immunotherapy

## Abstract

Prostate cancer is one of the most common malignancies in men and is frequently associated with tumor-promoting inflammation. Tumor-associated macrophages (TAMs) are known to facilitate cancer progression by suppressing antitumor immunity. Therefore, targeting TAMs represents a promising strategy for cancer therapy. This study aimed to investigate whether melittin-dKLA, a conjugated peptide consisting of melittin (MEL), which selectively binds M2-like macrophages, and the pro-apoptotic peptide d(KLAKLAK)_2_ (dKLA), can inhibit prostate cancer progression by targeting M2 macrophages. Human monocytic cells (THP-1 cells) were differentiated into TAMs using tumor-conditioned medium (TCM), and the conditioned medium from these TAMs was termed M-TCM. MEL-dKLA binding affinity was assessed using FITC-labeled melittin. A prostate cancer mouse model was established by subcutaneous injection of TRAMP-C2 cells, followed by MEL-dKLA administration every three days. As a result, THP-1-derived macrophages stimulated with TCM exhibited elevated expression of M2 markers (ARG1, CD206, and CD163). Prostate cancer cells (PC-3) stimulated with M-TCM showed increased proliferation and expression of epithelial-mesenchymal transition (EMT) markers. MEL-dKLA preferentially bound to M2 macrophages and TAMs, and inducing selective cytotoxicity. Conditioned media from MEL-dKLA-treated M2 macrophages and TAMs resulted in markedly decreased PC-3 cell proliferation, migration, and invasion. *In vivo*, MEL-dKLA treatment significantly reduced tumor growth, decreased the number of CD163^+^ M2 macrophages, and increased CD8^+^ T cell infiltration in tumor tissues. These findings demonstrate that MEL-dKLA suppresses prostate cancer progression by targeting M2-like TAMs both *in vitro* and *in vivo*. MEL-dKLA may serve as a promising therapeutic agent to modulate the tumor microenvironment in prostate cancer.

## 1 Introduction

Prostate cancer is one of the most prevalent malignancies among men and a leading cause of cancer-related mortality worldwide (1). While early-stage prostate cancer can often be managed effectively with surgery, radiotherapy, or androgen deprivation therapy, treatment options for advanced or castration-resistant prostate cancer remain limited and are associated with poor prognosis (2). Recent studies have highlighted the critical role of the tumor microenvironment (TME) in prostate cancer progression and therapeutic resistance, emphasizing the need for novel strategies that target not only tumor cells but also the supportive stromal and immune components of the TME (3, 4).

Among the various cellular components of the TME, tumor-associated macrophages (TAMs) have emerged as critical regulators in tumor progression (5). TAMs are primarily derived from circulating monocytes that infiltrate tumor tissues and differentiate in response to tumor-derived cues (6). These macrophages are broadly classified into two phenotypes: classically activated M1 and alternatively activated M2 phenotypes (7). Notably, M1 macrophages exhibit antitumor properties, whereas M2 macrophages are implicated in promoting tumor progression (8, 9). M2-polarized TAMs play immunosuppressive roles within the TME and facilitate tumor growth, invasion, and metastasis (10). In prostate cancer, high density of M2-like TAMs has been correlated with increased tumor aggressiveness and poor clinical prognosis (11). Given their pro-tumorigenic functions, M2-like TAMs represent a promising therapeutic target in prostate cancer (12). Strategies aimed at depleting TAMs, inhibiting their recruitment, or reprogramming them toward an M1-like phenotype have shown potential in preclinical models (13).

Melittin, a prominent cationic amphipathic peptide isolated from *Apis mellifera* (honeybee) venom, has garnered significant attention for its diverse biological activities, including antimicrobial, anti-inflammatory, and antineoplastic properties (14, 15). Recent investigations have elucidated a notable specificity in melittin’s interaction with macrophage subsets. Specifically, research indicates that melittin preferentially binds to M2-like macrophages (16, 17). In a previous study, we developed a peptide–drug conjugate, melittin-dKLA (MEL-dKLA), designed to target M2-like TAMs. MEL-dKLA consists of melittin conjugated to the pro-apoptotic peptide d(KLAKLAK)_2_ (dKLA) via a flexible linker. It was shown to selectively induce apoptosis in M2-like TAMs, leading to reduced tumor growth and enhanced antitumor immunity in melanoma and lung cancer models (18–20).

In this study, we investigate whether MEL-dKLA inhibits tumor progression by targeting M2-like TAMs within a prostate cancer model. Our findings aim to inform the development of novel immunomodulatory therapies targeting the TME in prostate cancer.

## 2 Materials and methods

### 2.1 Peptide

Melittin (GIGAVLKVLTTGLPALISWIKRKRQQ), dKLA (d[KLAKLAKKLAKLAK]), and MEL-dKLA (GIGAVLKVLTTGLPALISWIKRKRQQGGGGS-d[KLAKLAKKLAKLAK]) peptides were synthesized and purified to greater than 95% purity from GenScript (Piscataway, NJ, USA).

### 2.2 Cell cultures

THP-1 cells were purchased from the American Type Culture Collection (ATCC) and cultured according to their specific indications, using an RPMI 1640 medium supplemented with non-heat-treated 10% fetal bovine serum (FBS; WelGENE, Deagu, Korea), 2 mM L-glutamine, 0.05 mM β-mercaptoethanol, 10 mM HEPES, 4500 mg/L glucose, 100 U/ml penicillin and 100 μg/ml streptomycin (Gibco; Thermo Fisher Scientific, Inc., Waltham, MA, USA) at 37°C in a humidified 5% CO_2_ incubator. The human prostate cancer cell line (PC-3), obtained from the ATCC, were cultured in RPMI 1640 medium containing 2.05 mM L-glutamine, 2 g/liter sodium bicarbonate and 2 g/liter glucose (WelGENE) together with 10% FBS (WelGENE), 100 U/ml penicillin and 100 μg/ml streptomycin (Gibco) at 37 °C in a humidified 5% CO2 atmosphere. The medium exchange was performed every 2–3 days with reseeding at a density of 5 × 10^5^ cells/ml. The murine prostate cancer TRAMP C2 was maintained in DMEM media (Welgene, Gyeongsan, Korea) supplemented with 10% fetal bovine serum (FBS; Welgene, Gyeongsan, Korea) and 100U/ml penicillin and 100 μg/ml streptomycin (Invitrogen, CA, USA). The cells were incubated at 37°C, 95% humidity, and 5% CO2.

### 2.3 Differentiation of macrophages

THP-1 cells were differentiated into macrophages by incubating with 100 nM phorbol 12-myristate 13-acetate (PMA, Sigma-Aldrich, St. Louis, MO, USA) for 24 h. M1 macrophages were induced by incubating with 20 ng/ml of interferon-gamma (IFN-γ) (ProSpec, East Brunswick, NJ, USA) and 100 ng/ml of lipopolysaccharide (LPS) (Sigma-Aldrich). M2 macrophages were obtained by incubating with 20 ng/ml of interleukin-4 (IL-4) (ProSpec) and 20 ng/ml of interleukin-13 (IL-13) (ProSpec). TAMs were polarized by incubating with 20% conditioned medium derived from PC-3 cells.

### 2.4 Preparation of conditioned medium

To obtain conditioned media of tumor cells (TCM), PC-3 cells were seeded at 2 × 10^5^ cells/well in culture medium in 24‐well plates (Corning Inc., Kennebunk, ME, USA). After 24 h, the medium was replaced with serum‐free RPMI1640 medium, and the cells were incubated for 24 h. For conditioned media of macrophages, THP-1 cells were seeded at 2 × 10^5^ cells/well in culture medium in 24‐well plates (Corning Inc) and incubated with 100 nM PMA for 24 h. Cells were polarized into M0, M1, and M2 macrophages or TAM macrophages by TCM and changed to serum‐free RPMI1640 medium. After 24 h, the medium was changed to serum‐free RPMI1640 media, and the cells were incubated for 24 h. Supernatants were harvested and clarified with syringe filters (0.2 μm, Millipore Corp., Bedford, MA, USA).

### 2.5 Flow cytometry analysis

To determine the affinity of melittin to M2 macrophages, THP-1 cells were differentiated into M0, M1, M2 macrophages and TAMs. The cells were treated with 50 nM melittin conjugated with FITC for 1 h. To test the change of macrophage population in tumor tissue of prostate cancer mouse model, the single cells were isolated from tumor tissue through a 40 μm nylon mesh strainer after dissociation by DNase I (1 U/mL) and collagenase D (1 mg/ml). Cells were stained with anti-CD45, anti-F4/80, anti-CD86, anti-CD206, and anti-CD8 antibodies. For the Annexin V assay, THP-1 cells were differentiated into M0, M1, M2 macrophages. The cells were incubated with 1 µM MEL-dKLA for 1 hour at 37 °C. Cells were harvested and stained with APC-conjugated Annexin V (Invitrogen) for 15 min at room temperature. Following washing with BD Pharmingen Stain Buffer (BD biosciences), the cells were treated with 7-AAD Viability Staining Solution (Invitrogen) containing BD Pharmingen Stain Buffer. Data were analyzed using a BD FACS Canto II flow cytometer (BD biosciences, San Jose, CA, USA) and FlowJo software (BD biosciences).

### 2.6 Cell viability assay

THP-1 cells were differentiated into M2 macrophages using the method described above. The cells were treated with MEL-dKLA (1 μM) for 1 h, washed with PBS, and cultured in culture medium for 24 h. Cell viability was analyzed using the CCK‐8 assay: CCK‐8 reagent (Enzo Life Sciences, Farmingdale, NY, USA) was added to each well; incubation was continued for 2 h, and absorbance was measured at 450 nm with a microplate reader (Molecular Devices, San Jose, CA, USA).

### 2.7 ELISA assays

THP-1 cells were differentiated into M0, M1, M2 macrophages, and TAMs using the method described above. After differentiation, cells were treated with MEL-dKLA (1 μM) for 1 h, washed with PBS, and cultured in culture medium for 24 h. The supernatant of macrophages was collected. Markers of M2 macrophages such as IL-10 and TGF-β, and M1 macrophages such as IL-12 and CXCL10 were measured by ELISA kits according to the manufacturer’s instructions (BD Biosciences Inc.).

### 2.8 Real-time quantitative PCR

Total RNA was extracted using Easy-Blue reagent. Concentrations of RNA were determined and quantified by measuring absorbance at 260 and 280 nm with a spectrophotometer. Complementary DNA (cDNA) was synthesized from total RNA using a Maxime RT PreMix kit (Intron biotechnology, Seongnam, Korea). The sequences of the primers are shown in **Supplementary Table 1**. Real-time PCR analysis was performed with SYBR Green Master Mix. PCR conditions were forty-five cycles at 95˚C for 5 min, followed by 95˚C for 10 sec, 60˚C for 10 sec and 72˚C for 10 sec. mRNA expression were quantified in triplicate. Data were measured with CFX Software (Bio-Rad, Richmond, CA, USA). GAPDH and β-actin were used as internal controls.

### 2.9 Western blot analysis

Cells were harvested and lysed in PRO-PREP protein extraction solution (Intron biotechnology). Protein concentrations were measured with a Bradford Protein Assay Reagent kit (Bio-Rad, Richmond, CA, USA). Proteins were fractionated by 10% SDS-polyacrylamide gels electrophoresis (PAGE) and transferred onto polyvinylidene difluoride (PVDF) membranes. These were incubated with anti-E-cadherin, anti-fibronectin, anti-vimentin, anti-PCNA, anti-TGF-β, and anti-β-actin Ab as primary antibodies. Goat anti-rabbit horseradish peroxidase-conjugated IgG or goat anti-mouse horseradish peroxidase-conjugated IgG (Abcam, Cambridge, MA, USA) served as secondary antibodies. Protein bands were visualized using a chemiluminescence detection kit (SurModics, Eden Prairie, MN, USA).

### 2.10 Wound healing assay

We assessed the migration of prostate cancer using wound healing assays. PC-3 cells were seeded at 2 × 10^5^ cells/well in 24-well plates and cultured in RPMI1640 with 10% FBS. When the cells reached confluence, they were wounded by scraping across the surface of the well with a sterile micropipette tip. The cells were immediately washed, and the wells were filled with serum-free medium or 20% conditioned media of M0, M1, M2 macrophages, and M-TCM without or with MEL-dKLA and incubated or 24 hr. Before and after incubation at least five different fields of the wounded area of each sample were photographed using an inverted microscope (Olympus, Tokyo, Japan). Wound areas were measured with ImageJ software (NCI, Bethesda, MD, USA). The percent of each wounded area filled by cell migration was calculated as: (mean wounded breadth–mean remaining breadth)/mean wounded breadth × 100.

### 2.11 Invasion assay

The invasiveness of prostate cancer cells treated with conditioned media of macrophages was tested according to the manufacturer’s instructions for the invasion assay (Corning Inc.) with slight modifications. Briefly, invasiveness was assessed using 24‐well plates fitted with polycarbonate 8‐μm pore membrane inserts (Corning Inc.) pre‐coated with Matrigel (200‐300 μg/mL) for 2 h at 37 °C. The lower wells were filled with 350 μL of serum‐free RPMI1640 medium or 20% conditioned medium (conditioned media of M0, M1, M2, and M-TCM without or with MEL-dKLA). The upper wells were filled with 200 μL PC-3 cells (5 × 10^4^ cells/well) in serum‐free medium. The plates were incubated for 24 h. The cells were then fixed in methanol and stained with Giemsa. Five randomly selected fields per membrane were counted under a light microscope (Olympus). The invasion index was calculated from the number of cells that migrated in response to conditioned medium compared with the control without conditioned medium.

### 2.12 Animal study

Male C57BL/6J of 6–8 weeks were obtained from DBL (Chungbuk, South Korea). All mice were maintained under specific pathogen-free conditions on a 12-h light/dark cycle with free access to food and water. To establish a mouse model of prostate cancer, TRAMP C2 cells were mixed with Matrigel (Corning, NY, USA) and subcutaneously injected into the right flank (1×10^5^ cells/mice). One week after tumor cells injection, Melittin, dKLA, and MEL-dKLA (200 nmol/mice) were intraperitoneally injected every 3 days. Tumor volume was measured using a digital caliper every 3 days. The calculation formula of tumor volume is V = (width × width ×length)/2. All mice were euthanized when the tumor volume reached 1000mm^3^. Animal studies were approved by the Institutional Animal Care and Use Committee of Kyung Hee University (KHUASP(SE)-20-382).

### 2.13 Immunohistochemistry

Tumor tissues were fixed overnight in 10% neutral buffered formalin and cut into 4 μm of regular thickness after being embedded in paraffin. The sections were dipped in xylene and then 100%, 90%, 80%, and 70% ethanol, and washed under running tap water for rehydration. Tissue sections were washed with deionized water for 10 min after bleaching. The tissue antigen was heat-retrieved using sodium citrate buffer (pH 6.0) for 10 min in a microwave. The tissue was incubated with 3% H2O2 for 15 min and blocked with 5% BSA containing 0.1% Tween20 for 1 h. The slides were incubated with primary antibodies of F4/80, CD80, CD163, CD8, E-cadherin (Cell Signaling Technology), Vimentin, and Ki67 (Abcam) for overnight. And then, the slides were incubated using a VECTASTAIN Elite ABC kit (VECTOR Laboratories, Burlingame, California, USA) and visualized with a DAB Substrate kit (VECTOR Laboratories). All antibodies were diluted in a 5% BSA solution. Five or more random fields of protein expression in the tumor tissues were detected using an optical microscope.

### 2.14 Statistical analysis

All data are expressed as means ± standard deviations of three or four independent experiments. Comparisons were made using the two-tailed Mann-Whitney U-test, one-way and Two-way ANOVA (GraphPad Software Inc., CA, USA), and *P*-values < 0.05 were considered statistically significant.

## 3 Results

### 3.1 M2 polarization of macrophages induced by tumor-conditioned medium

**Figure 1:**
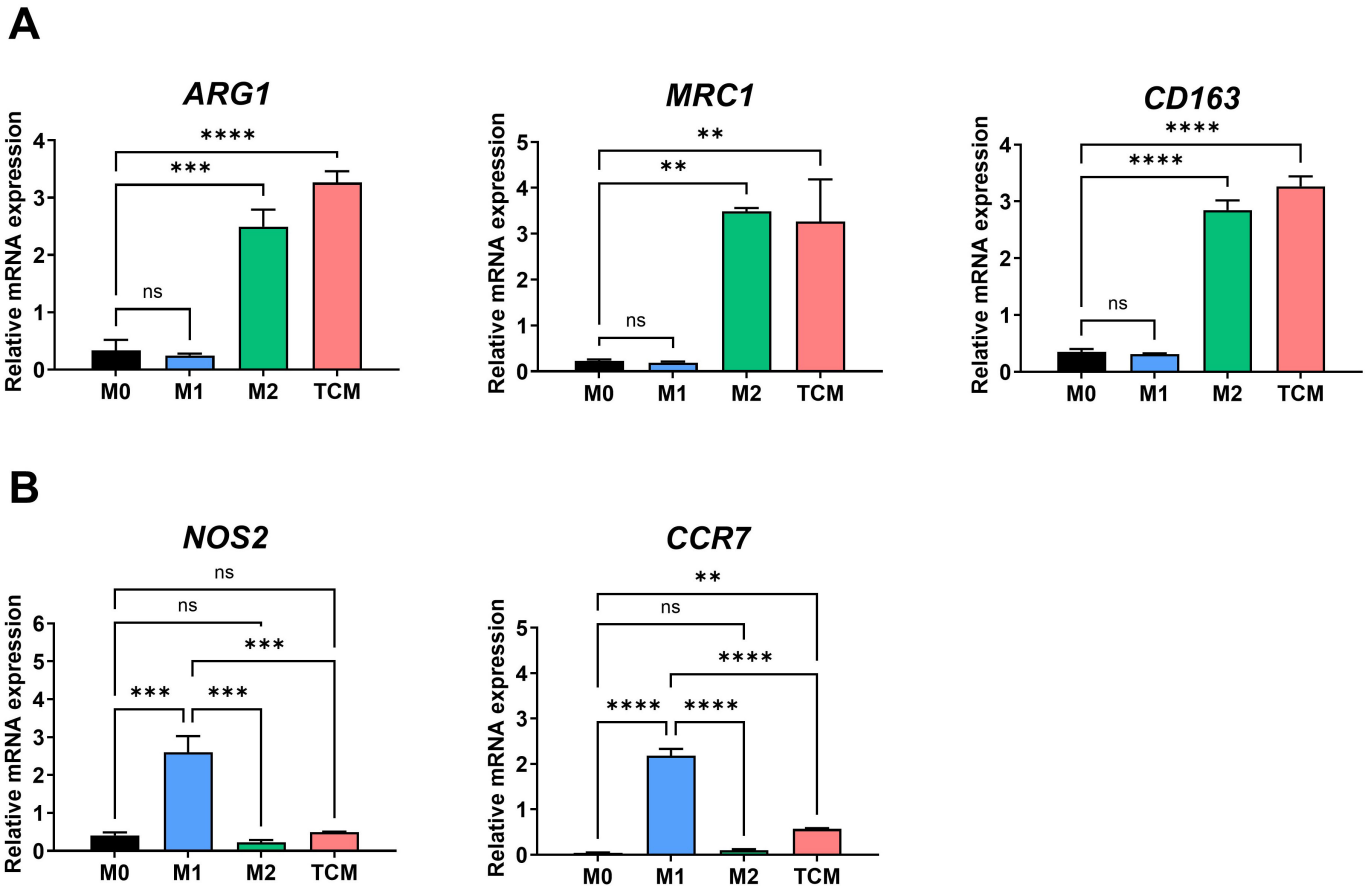
M2-like polarization of THP-1-derived macrophages by tumor-conditioned medium. THP-1 monocytes were differentiated into macrophages (M0) with 100 nM PMA for 24 h, followed by polarization into M1 macrophages using 100 ng/ml LPS and 20 ng/ml IFN-γ, or into M2 macrophages using 20 ng/ml IL-4 and 20 ng/ml IL-13 for 72 hours TAMs were generated by culturing macrophages with 20% TCM from PC-3 prostate cancer cells. **(A)** Expression levels of M2-associated genes (*ARG1, CD206, CD163*) were analyzed by qRT-PCR. **(B)** Expression levels of M1-associated genes (*NOS2, CCR7*) were also assessed by qRT-PCR. Data are shown as relative mRNA expressions normalized to GAPDH. **p < 0.01; ***p < 0.001; ****p < 0.0001; ns, not significant.

Tumor cells are known to induce macrophage polarization toward the M2 phenotype, and TAMs in the TME are predominantly of the M2 type (21). To assess whether prostate cancer cells promote M2 polarization of macrophages, THP-1-derived macrophages were treated with TCM obtained from PC-3 cells. Macrophages treated with TCM exhibited significantly elevated mRNA levels of M2 markers (*ARG1, MRC1*, and *CD163*), while expression of M1 markers (*NOS2* and *CCR7*) was markedly reduced compared to M1 macrophages (**Figures 1A, B**). These findings indicate that prostate cancer cell-derived TCM promotes the polarization of THP-1-derived macrophages into M2-like TAMs.

### 3.2 Induction of proliferation, migration, and epithelial-mesenchymal transition in prostate cancer cells by conditioned medium of TAMs

**Figure 2:**
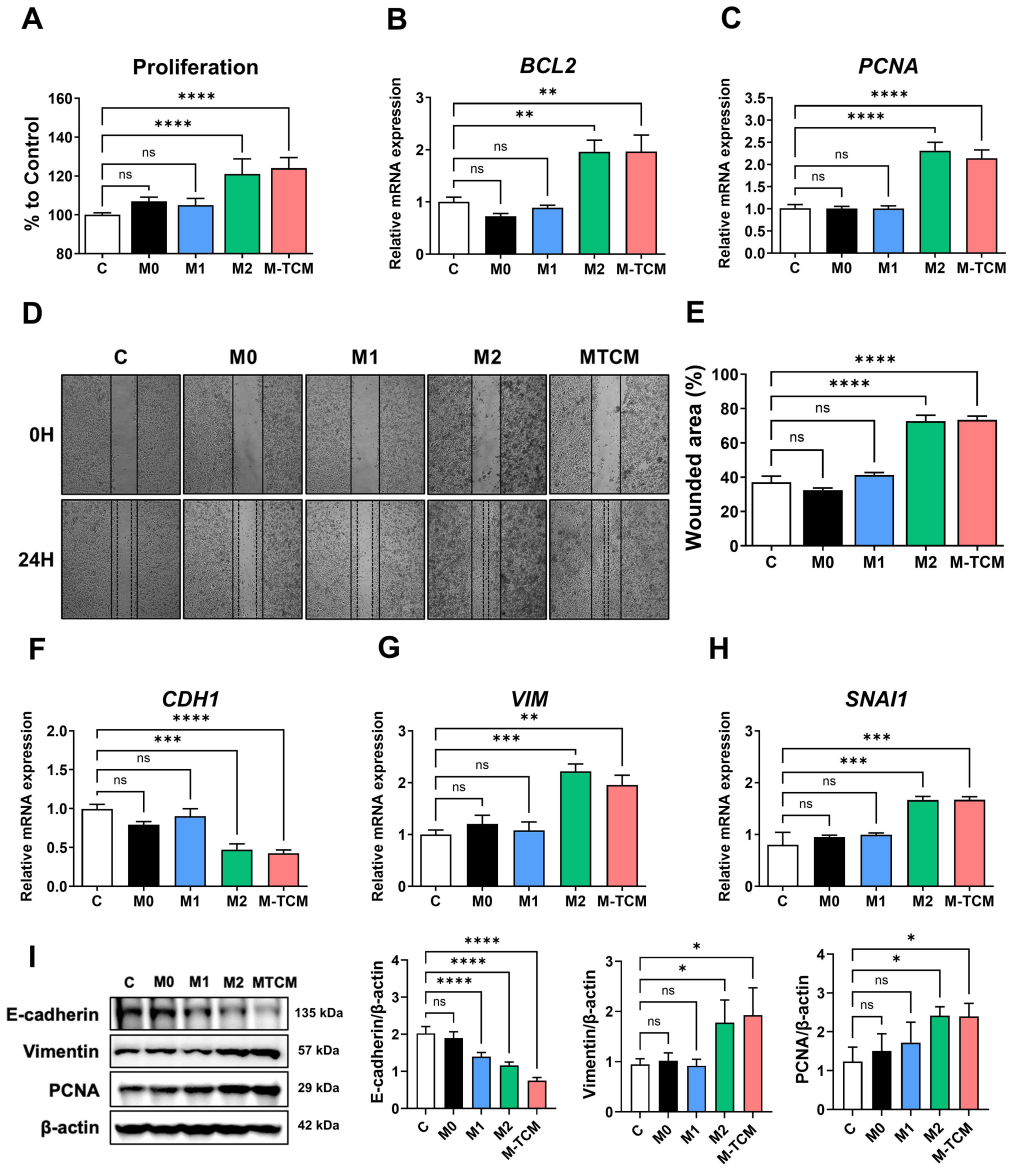
Induction of proliferation, migration, and epithelial-mesenchymal transition into prostate cancer cells by conditioned medium of TAMs. **(A)** PC-3 cells were treated with 20% conditioned medium (CM) from M0, M1, M2 macrophages, or TAMs (M-TCM) for 24 hours, and cell proliferation was measured using the CCK-8 assay. **(B, C)** Relative mRNA expression levels of proliferation-associated genes BCL2 **(B)** and PCNA **(C)** were assessed by qRT-PCR. **(D)** Representative images of wound healing assays performed at 0 and 24 hours after scratch in PC-3 cells treated with each type of CM. **(E)** Quantification of wounded area (%) was calculated using ImageJ software. **(F–H)** mRNA expression of epithelial and mesenchymal markers in PC-3 cells treated with each type of CM, assessed by qRT-PCR: *CDH1* **(F)**, *VIM* **(G)**, and *SNAI1* **(H)**. **(I)** Protein expression of E-cadherin, Vimentin, and PCNA analyzed by Western blot. GAPDH was used as a loading control. *p < 0.05; **p < 0.01; ***p < 0.001; ****p < 0.0001; ns, not significant.

In the TME, M2-like TAMs play a pivotal role in promoting tumor progression, including enhanced proliferation and invasion of various cancers (5). To determine the functional effects of M2 macrophages and TAMs on prostate cancer progression, PC-3 cells were treated with conditioned media from M0, M1, M2 macrophages, or TAMs (M-TCM). Cell proliferation was significantly enhanced in PC-3 cells treated with M2 and M-TCM compared to the control (**Figure 2A**). Consistently, the expression of proliferation-related genes *BCL2* and *PCNA* was significantly upregulated in these groups (**Figures 2B, C**). To evaluate the impact on migratory potential, a wound healing assay was performed. PC-3 cells treated with M2 and M-TCM exhibited markedly enhanced wound closure at 24 hours compared to control, indicating increased migration capacity (**Figures 2D, E**). Furthermore, epithelial-mesenchymal transition (EMT) was assessed by analyzing the expression of EMT-related markers. Treatment with M2 and M-TCM led to a significant reduction in *CDH1* (E-cadherin) expression as an epithelial cell marker, while *VIM* (Vimentin) and *SNAI1* (Snail1) as a mesenchymal marker were significantly upregulated (**Figures 2F–H**). These gene expression changes were supported by protein expression level, which showed decreased E-cadherin and increased Vimentin and PCNA (**Figure 2I**). Thus, these results demonstrate that M2 macrophages and TAMs promote proliferation, migration, and EMT in prostate cancer cells, thereby contributing to a more aggressive tumor phenotype.

### 3.3 Targeting of M2-like tumor-associated macrophages by MEL-dKLA

**Figure 3:**
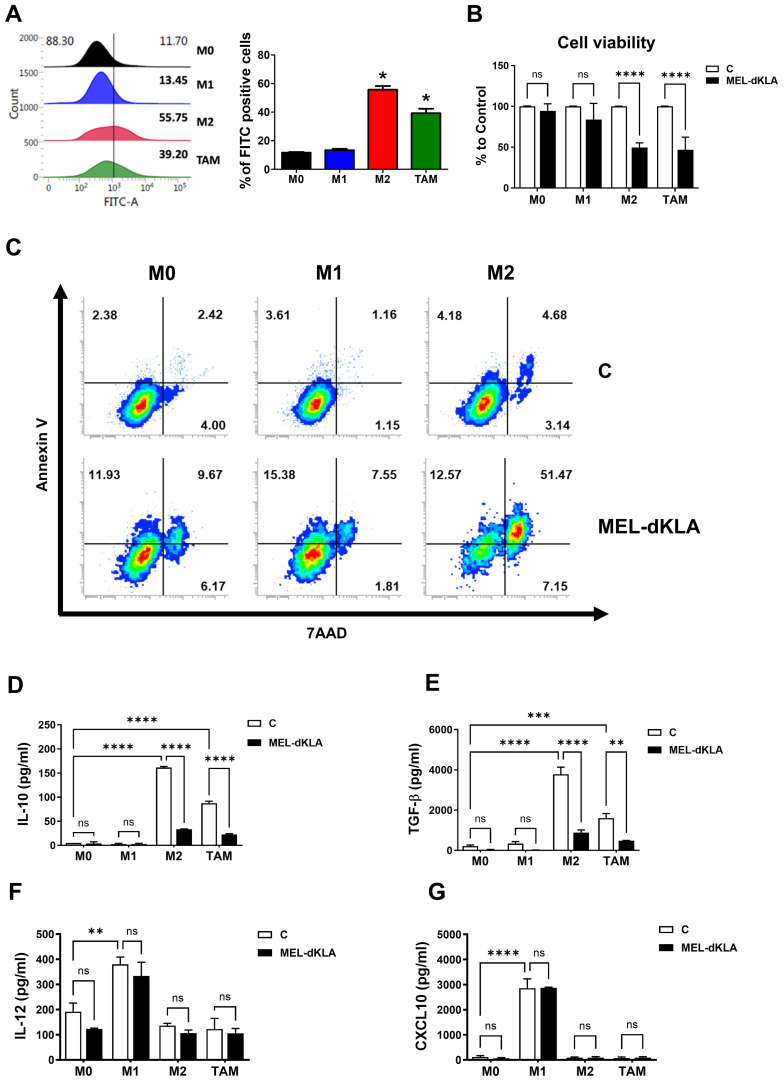
Targeting M2-like tumor-associated macrophages by MEL-dKLA. **(A)** THP-1-derived macrophages (M0, M1, M2, TAM) were treated with FITC-conjugated melittin (50 nM), and FITC-positive cells were quantified by flow cytometry to assess binding affinity. **(B)** Cell viability was analyzed using the CCK-8 assay. **(C)** Annexin V/7-AAD double staining was performed, and stained cells were analyzed using flow cytometry. **(D–G)** Macrophages were treated with MEL-dKLA or control (media only). Cytokine levels were measured using ELISA: IL-10 **(D)**, TGF-β **(E)**, IL-12 **(F)**, and CXCL10 **(G)**. *p < 0.05; **p < 0.01; ***p < 0.001; ****p < 0.0001; ns, not significant.

To evaluate the binding specificity of melittin toward M2-like TAMs, FITC-conjugated melittin was synthesized and applied to THP-1-derived macrophages polarized into M0, M1, M2, and TAM phenotypes. Flow cytometric analysis showed that melittin exhibited significantly higher binding affinity to M2 macrophages and TAMs compared to M0 and M1 macrophages (**Figure 3A**). MEL-dKLA exhibited enhanced cytotoxicity against M2 macrophages, with an IC_50_ of 0.577 μM, compared to melittin alone (IC_50_ = 4.437 μM), while dKLA showed no cytotoxicity (**Supplementary Figure 1**). To determine whether MEL-dKLA induces selective cytotoxicity, each macrophage subtype was treated with MEL-dKLA. The viability of M2 macrophages and TAMs was significantly decreased, whereas M0 and M1 macrophages remained unaffected (**Figure 3B**). On treatment with MEL-dKLA, the number of apoptotic cells was significantly increased in M2 macrophages, compared to M0 and M1 macrophages (**Figure 3C**). We next examined the impact of melittin-dKLA on cytokine production. MEL-dKLA significantly reduced the production of anti-inflammatory cytokines IL-10 and TGF-β in M2 macrophages and TAMs (**Figures 3D, E**), while no significant change was observed in M0 or M1 macrophages. In contrast, the production of the pro-inflammatory cytokines IL-12 and CXCL10 was not increased in M2 and TAMs. Notably, MEL-dKLA treatment resulted in unchanged IL-12 and CXCL10 levels in M1 macrophages (**Figures 3F, G**). These results suggest that MEL-dKLA selectively targets M2-like TAMs by inducing cytotoxicity and suppressing anti-inflammatory cytokine production, without activating pro-inflammatory responses in M0 and M1 subtypes.

### 3.4 Inhibition of proliferation, migration, and invasion of prostate cancer cells by conditioned medium of MEL-dKLA-treated TAMs

**Figure 4:**
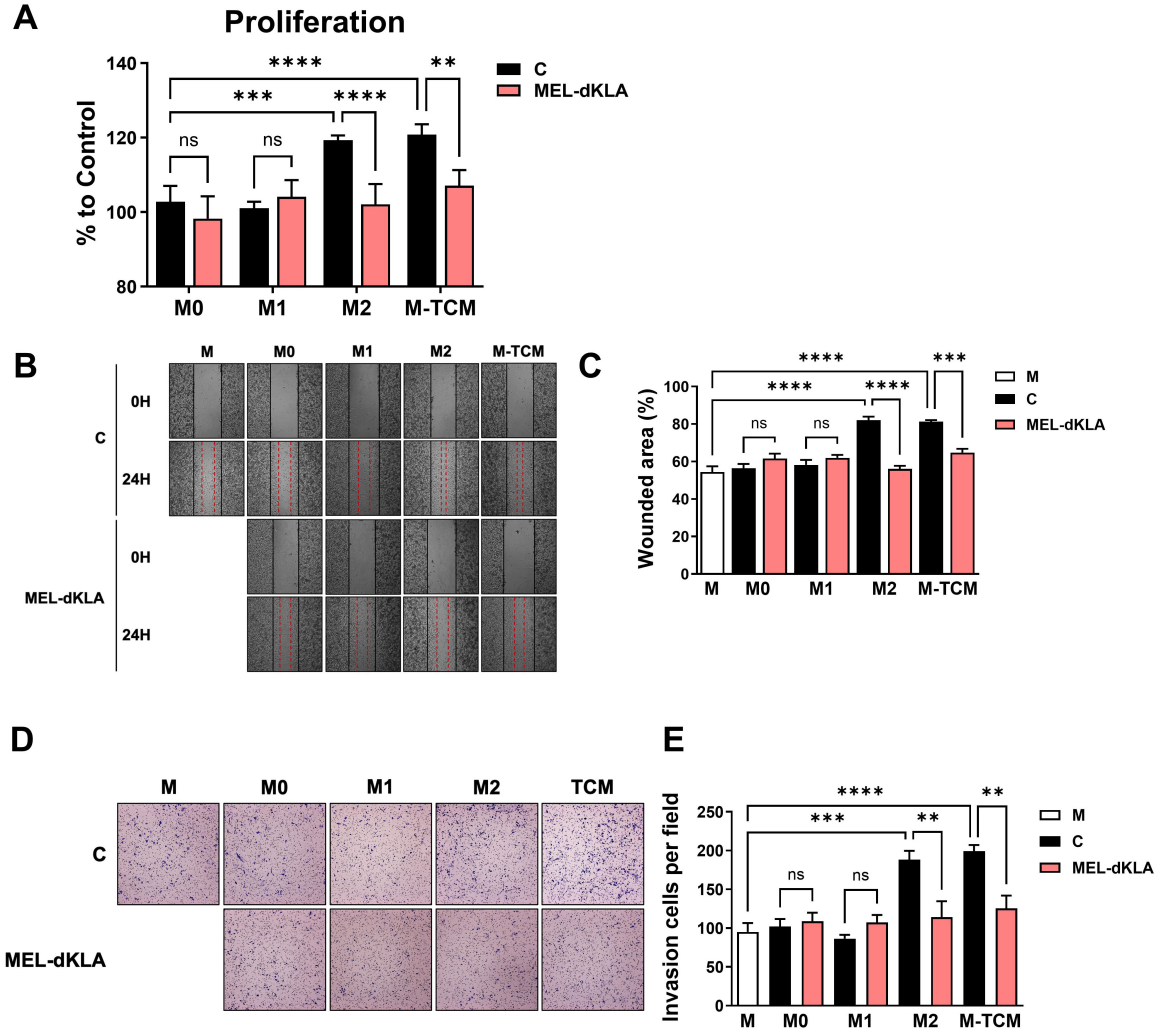
Inhibition of proliferation, migration, and invasion of prostate cancer cells by conditioned medium of MEL-dKLA-treated TAMs. **(A)** PC-3 cells were cultured in conditioned medium derived from M0, M1, M2 macrophages, and TAMs, with or without MEL-dKLA. Cell proliferation was measured using the CCK-8 assay. **(B)** Representative images of wound healing assays at 0 and 24 hours after scratch injury in PC-3 cells treated with conditioned medium from each macrophage types. **(C)** Quantification of wounded area (%) using ImageJ software. **(D)** Representative images from transwell invasion assays showing PC-3 cell invasion after 24 hours **(E)** Number of invaded PC-3 cells per field was quantified from five randomly selected microscopic fields. **p < 0.01; ***p < 0.001; ****p < 0.0001; ns, not significant.

To determine whether MEL-dKLA inhibits the proliferation and migration of prostate cancer cells, PC-3 cells were treated with conditioned medium derived from M0, M1, M2 macrophages, and TAMs, with or without MEL-dKLA pretreatment. The PC-3 cell proliferation was significantly enhanced by conditioned medium of M2 macrophages (M2) and TAMs (M-TCM), while pretreatment of these macrophages with MEL-dKLA significantly inhibited this proliferation effect (**Figure 4A**). Additionally, PC-3 cells exhibited increased migration when treated with conditioned medium from M2 and TAMs, but this effect was markedly reduced in the conditioned medium obtained from MEL-dKLA-pretreated M2 macrophages and TAMs (**Figures 4B, C**). To evaluate the effect of MEL-dKLA on the invasion of prostate cancer cells, the invasion capacity was assayed using a Matrigel-coated transwell. PC-3 cells were seeded in the upper chamber and the conditioned medium of M0, M1, M2 macrophages, and TAMs, either untreated or pretreated with MEL-dKLA was filled in the lower chamber. M2 and M-TCM significantly increased the number of invaded cells, whereas MEL-dKLA pretreatment of these macrophages led to a significant reduction in PC-3 cell invasion (**Figures 4D, E**). These results suggest that MEL-dKLA attenuates the tumor-promoting activity such as proliferation, migration, and invasion by targeting M2-like TAMs.

### 3.5 Suppression of prostate tumor growth by MEL-dKLA *in vivo*

**Figure 5:**
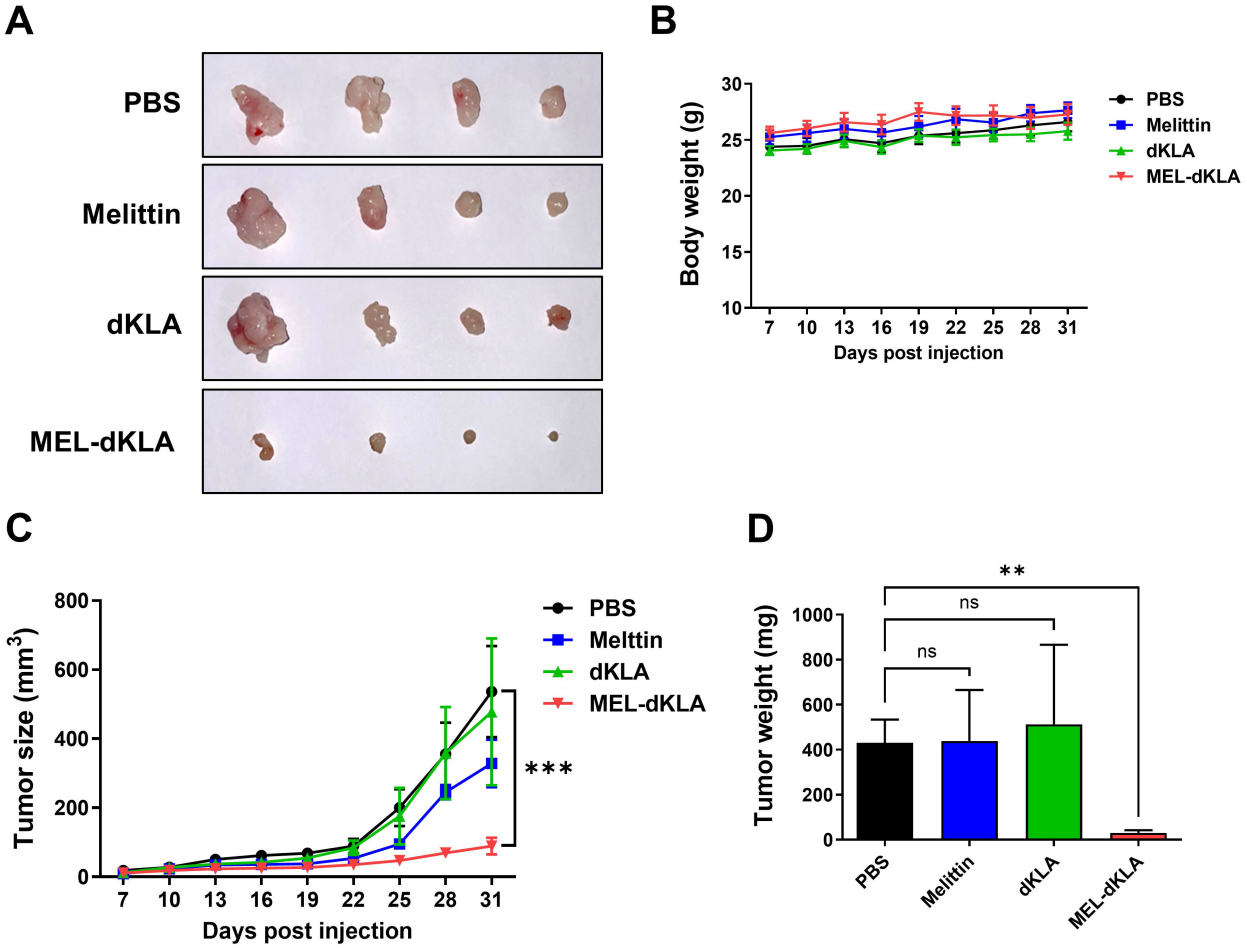
Suppression of prostate tumor growth by MEL-dKLA *in vivo*. **(A)** Representative images of tumor tissues collected from mice injected intraperitoneally with PBS, melittin, dKLA, or MEL-dKLA every 3 days, starting 7 days after TRAMP-C2 cell implantation. **(B)** Body weights of mice were measured throughout the treatment period to assess systemic toxicity. **(C)** Tumor volumes were measured every 3 days using calipers and calculated using the formula: (width² × length)/2. **(D)** Tumors were excised on day 31, and final tumor weights were recorded. Data are presented as mean ± SD (n = 4 per group). Statistical analysis was performed using two-way ANOVA for tumor growth and one-way ANOVA with Tukey’s *post hoc* test for tumor weight. **p < 0.01; ***p < 0.001; ns, not significant.

To evaluate the anti-tumor efficacy of MEL-dKLA *in vivo*, a subcutaneous prostate cancer model was established by injecting TRAMP-C2 cells into the right flank of C57BL/6 mice. Mice were administrated by intraperitoneal injections of PBS, melittin, dKLA, or MEL-dKLA every 3 days. Tumor volume demonstrated that MEL-dKLA markedly reduced tumor growth compared to control groups (**Figures 5A, C**). Body weight remained stable in all groups throughout the treatment period, indicating no overt systemic toxicity (**Figure 5B**). Tumor weights were significantly lower in the MEL-dKLA group compared to PBS groups (**Figure 5D**). These findings indicate that MEL-dKLA effectively suppresses prostate tumor growth *in vivo* without apparent toxicity, suggesting therapeutic potential through targeting M2-like TAMs.

### 3.6 Inhibition of EMT and proliferation markers by MEL-dKLA in tumor tissues

**Figure 6:**
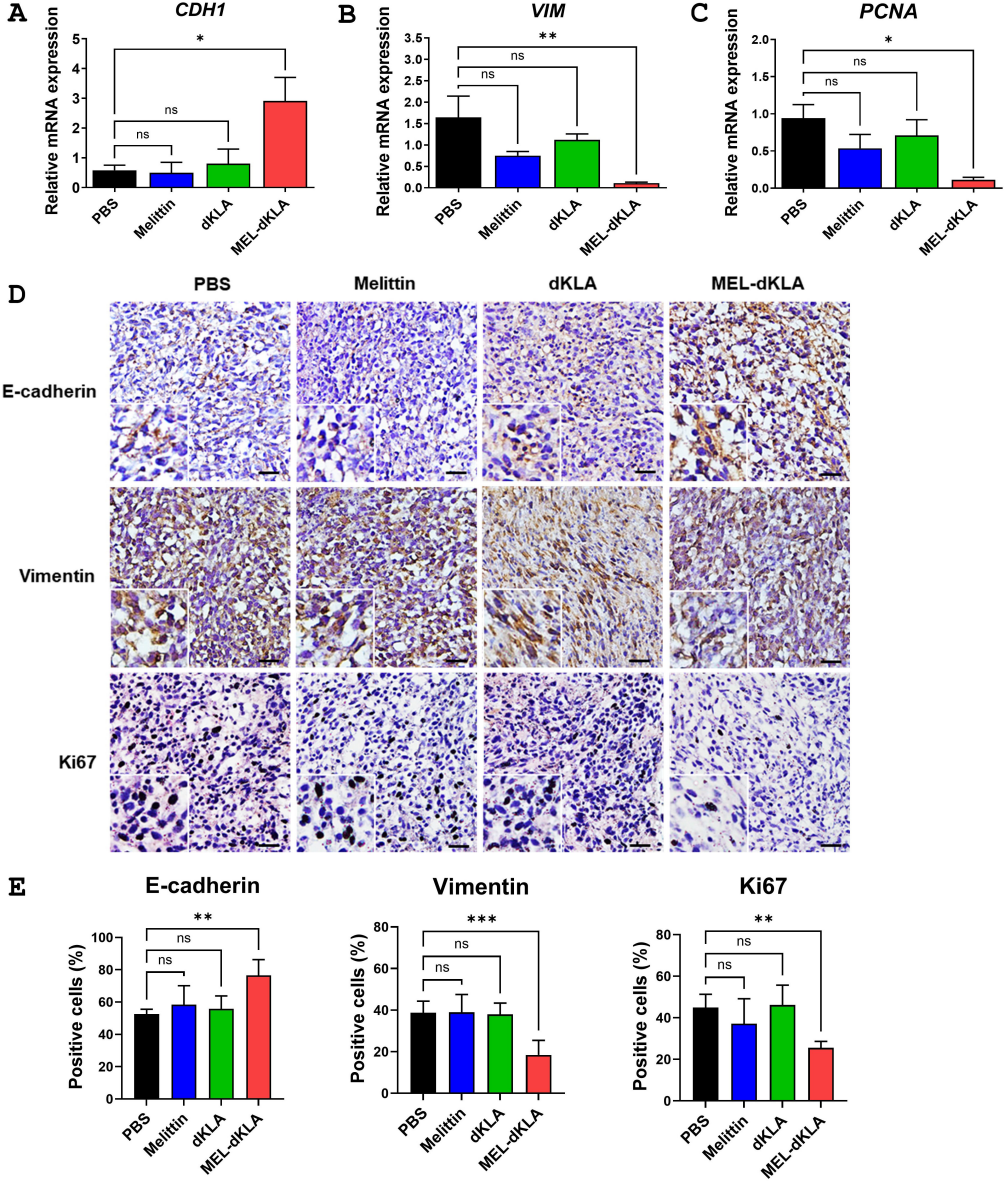
Inhibition of EMT and proliferation markers by MEL-dKLA in tumor tissues. **(A–C)** Relative mRNA expression of *CDH1* **(A)**, *VIM* **(B)**, and *PCNA* **(C)** in tumor tissues collected from PBS-, melittin-, dKLA-, or MEL-dKLA–treated mice were measured by qRT-PCR. **(D)** Representative images of immunohistochemical staining for E-cadherin, vimentin, and Ki67 in tumor sections from each treatment group. Scale bars: 100 μm. **(E)** Quantification of IHC-positive cells for E-cadherin, Vimentin, and Ki67 from five random fields per sample. Data are presented as mean ± SD from n = 4 mice per group. Statistical analysis was performed using one-way ANOVA followed by Tukey’s *post hoc* test. *p < 0.05; **p < 0.01; ***p < 0.001; ns, not significant.

To examine whether MEL-dKLA affects EMT and tumor cell proliferation *in vivo*, expression of EMT and proliferation markers was assessed in tumor tissues collected from the prostate cancer mouse model. Expression of the epithelial marker *CDH1* was significantly upregulated in tumors from MEL-dKLA-treated mice, whereas the mesenchymal marker *VIM* and the proliferation marker *PCNA* were significantly downregulated compared to control groups (**Figures 6A–C**). Consistent with these results, immunohistochemical (IHC) staining showed increased E-cadherin expression and decreased Vimentin and Ki67 expression in the MEL-dKLA-treated group compared to PBS groups (**Figures 6D, E**). In protein expression level of tumor tissues, MEL-dKLA treatment increased the expression of E-cadherin while reducing the levels of Fibronectin, PCNA, and TGF-β compared to control groups (**Supplementary Figure 2**). These findings indicate that MEL-dKLA suppresses EMT and cell proliferation *in vivo*, contributing to its anti-tumor efficacy.

### 3.7 Selective depletion of M2-like tumor-associated macrophages by MEL-dKLA treatment

**Figure 7:**
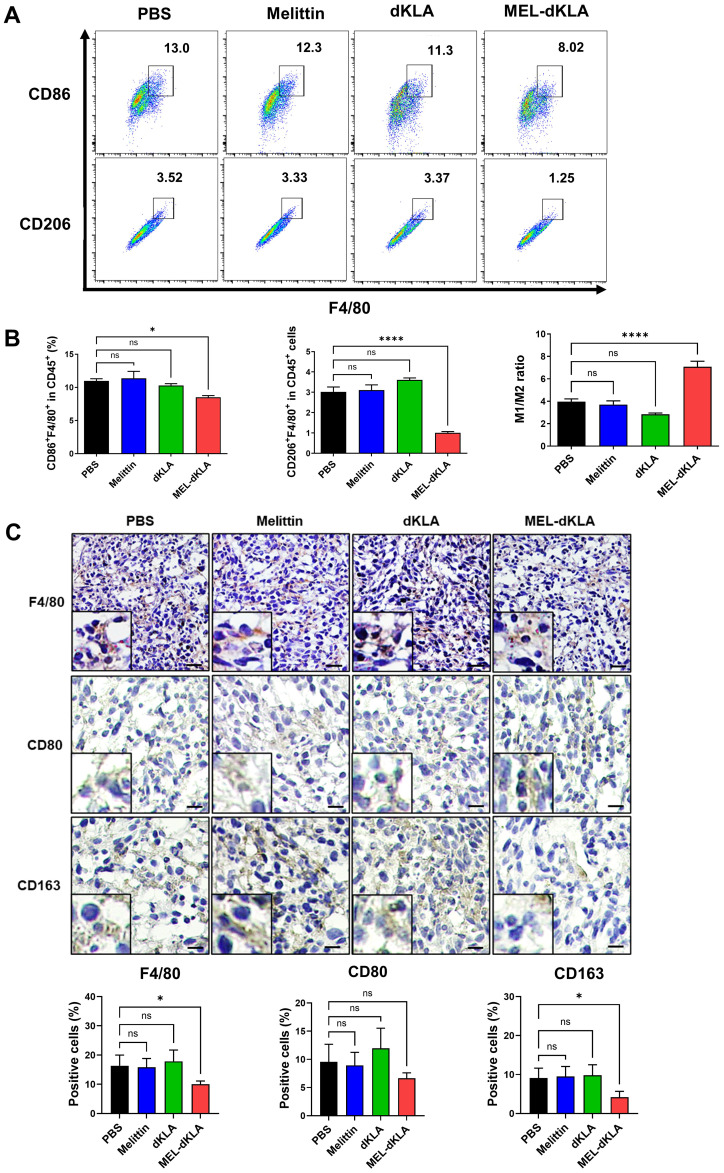
Selective depletion of M2-like TAMs by MEL-dKLA in the TME. **(A)** Flow cytometric analysis of CD86^+^F4/80^+^ M1 and CD206^+^F4/80^+^ M2 macrophage populations among CD45^+^ cells isolated from tumor tissues of mice treated with PBS, melittin, dKLA, or MEL-dKLA. **(B)** Quantification of CD86^+^F4/80^+^, CD206^+^F4/80^+^, and the M1/M2 ratio in tumor-infiltrating immune cells. **(C)** Representative immunohistochemical staining of tumor sections for F4/80, CD80, and CD163. Scale bars: 50 μm. Insects show high-magnification images. Data are presented as mean ± SD (n = 4 mice/group). Statistical analysis was performed using one-way ANOVA with Tukey’s *post hoc* test. *p < 0.05; ****p < 0.0001; ns, not significant.

To evaluate whether MEL-dKLA selectively targets M2-like TAMs in TME, we analyzed the phenotype of tumor-infiltrating macrophages in tumor tissues by flow cytometry and immunohistochemistry. The proportion of CD206^+^F4/80^+^ M2 macrophages was significantly reduced in the MEL-dKLA-treated group (3.52% in PBS vs. 1.25% in MEL-dKLA), whereas the proportion of CD86^+^F4/80^+^ M1 macrophages showed a slight compared to control groups (13.0% vs. 8.02%). As a result, the M1/M2 ratio was markedly increased, not due to M1 enhancement but rather due to preferential depletion of the M2 phenotype (**Figures 7A, B**). Consistently, immunohistochemical staining revealed a significant decrease in F4/80^+^ total macrophages and CD163^+^ M2 macrophages in MEL-dKLA–treated group, while the number of CD80^+^ M1 macrophages remained unchanged (**Figure 7C**). These findings suggest that MEL-dKLA does not broadly activate or expand M1 macrophages, but rather eliminates immunosuppressive M2-like TAMs, thereby reprogramming the TME toward a less immunosuppressive state.

### 3.8 Enhancement of CD8^+^ T cell infiltration by MEL-dKLA treatment

**Figure 8:**
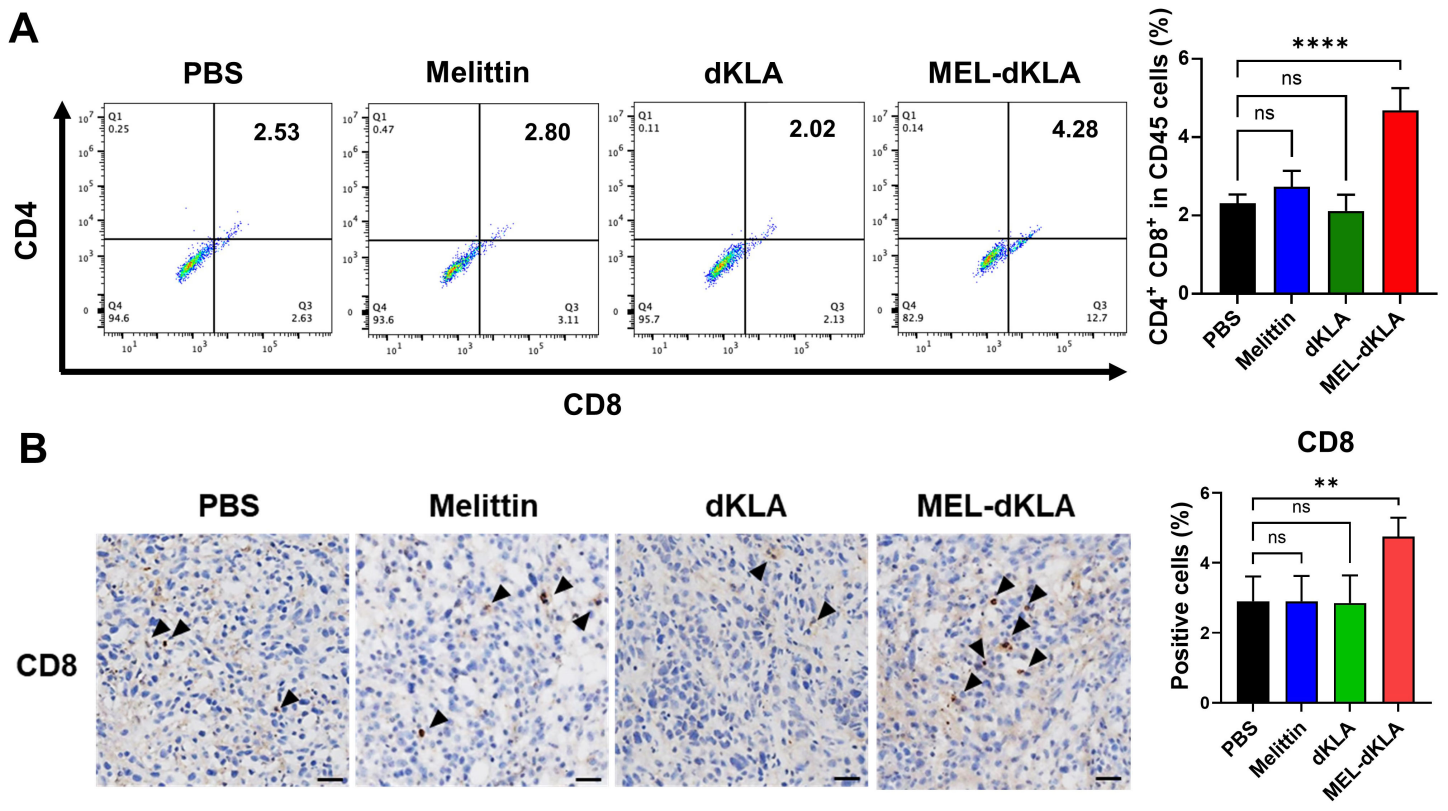
Enhancement of CD8^+^ T cell infiltration by MEL-dKLA treatment. **(A)** Flow cytometric analysis of CD8^+^CD45^+^ T cells in tumors tissues after treatment with PBS, melittin, dKLA, or MEL-dKLA. Percentages of CD8^+^ cells in CD45^+^ populations are shown (left), and quantification is presented (right). **(B)** Representative immunohistochemical staining of tumor sections for CD8^+^ T cells (arrowheads) from each treatment group. Scale bars: 100 μm. Bar graph (right) shows quantification of CD8^+^ T cell infiltration based on five randomly selected fields per section. Data are presented as mean ± SD (n = 4 mice/group). Statistical analysis was performed using one-way ANOVA with Tukey’s *post hoc* test. **p < 0.01; ****p < 0.0001; ns, not significant.

To determine whether MEL-dKLA modulates anti-tumor immune responses in the TME, we analyzed the infiltration of CD8^+^ T cells in tumor tissues. The proportion of CD8^+^CD45^+^ T cells was significantly increased in the MEL-dKLA–treated group compared to PBS groups (**Figure 8A**). Consistent with the flow cytometry results, immunohistochemical staining of tumor sections showed a significantly increased number of CD8^+^ T cells in the MEL-dKLA–treated group (**Figure 8B**). These results suggest that MEL-dKLA treatment enhances CD8^+^ T cell infiltration into the TME by reduction of M2-like TAMs, potentially contributing to its anti-tumor activity by reversing immunosuppression.

## 4 Discussion

Tumor-associated macrophages (TAMs), particularly the M2-like phenotype, play a pivotal role in the progression of prostate cancer by promoting tumor growth, angiogenesis, and immunosuppression. Targeting these cells has emerged as a promising strategy (22). In this study, we evaluated the efficacy of MEL-dKLA in selectively targeting M2-like TAMs to inhibit prostate cancer progression.

In the TME, tumor cells actively modulate their surroundings to create an immunosuppressive and pro-tumorigenic environment, notably by inducing the differentiation of macrophages into the M2-like phenotype (13). This phenomenon is mediated by various tumor-derived factors, including cytokines (e.g., IL-4, IL-10, TGF-β) and chemokines, which collectively promote M2 polarization of macrophages. In this study, tumor-conditioned medium derived from PC-3 cells induced M2 macrophage polarization, as evidenced by increased expression of M2 phenotype markers such as *Arg1*, *CD206*, and *CD163* (**Figure 1**). Furthermore, these polarized M2 TAMs promoted the proliferation and migration of PC-3 cells (**Figure 2**), suggesting a feed-forward loop that supports tumor aggressiveness. Therefore, these findings demonstrate the functional importance of M2-like TAMs in the TME and their potential as therapeutic targets for prostate cancer.

Recently, the field of cancer immunotherapy has increasingly focused on targeting immunosuppressive components within the TME, particularly M2-like TAMs, due to their critical role in tumor progression and resistance to therapy (23, 24). Several therapeutic strategies, including antibody-, nanoparticle-, and peptide-based agents, have been studied to deplete M2 macrophages or reprogram them into an inflammatory M1-like phenotype (25, 26). Among them, melittin, a major component of bee venom, has been studied to selectively bind to M2 macrophages and has potential as a drug delivery ligand (20, 27). In this study, melittin showed high binding to M2 macrophages and TAMs differentiated by PC-3-conditioned medium (TCM). Furthermore, MEL-dKLA, which is combined with a pro-apoptotic peptide, dKLA, induced apoptosis by preferentially binding to M2 macrophages and TAMs, while not affecting M0 or M1 macrophages (**Figure 3**). This selectivity is critical, as it enables the elimination of tumor-promoting macrophages while preserving those that contribute to antitumor immunity.

The escalating incidence and mortality associated with prostate cancer progression, particularly its transition from localized disease to aggressive, metastatic forms, underscores the urgent need for novel therapeutic strategies (28–30). M2-polarized TAMs are recognized as key drivers of tumor growth, angiogenesis, metastasis, and immune evasion within the TME (5, 24). Numerous studies have consistently demonstrated that elevated M2 infiltration in prostate cancer tissues correlates with poor prognosis, increased tumor growth, and enhanced metastatic potential (31, 32). Furthermore, M2 macrophages have been shown to directly contribute to EMT in prostate cancer cells, a crucial process facilitating tumor invasiveness and metastasis (33). This is often characterized by a decrease in epithelial cell markers like E-cadherin and an increase in mesenchymal markers such as Vimentin and N-cadherin, indicative of enhanced migratory and invasive capabilities in cancer cells (34). In our findings, the conditioned medium from MEL-dKLA-treated M2 macrophages and TAMs significantly reduced proliferation, migration, and invasion of PC-3 cells (**Figure 4**). *In vivo* studies using a TRAMP-C2 prostate cancer mouse model further corroborated these findings. MEL-dKLA treatment led to a marked reduction in tumor volume and weight without affecting the overall health of the animals, as evidenced by stable body weights (**Figure 5**). Additionally, the expression of EMT markers and proliferation-associated proteins such as PCNA and TGF-β were reduced, indicating a reversion of aggressive tumor phenotypes (**Figure 6**).

Accumulating evidence suggests that M2-like TAMs play a central role in establishing an immunosuppressive TME that impairs anti-tumor immunity, particularly by inhibiting cytotoxic CD8^+^ T cell infiltration and function (35, 36). In prostate cancer, the presence of M2 macrophages is associated with lower CD8^+^ T cell density, enhanced tumor progression, and poor clinical prognosis (22, 37). Our findings demonstrate that MEL-dKLA treatment selectively depletes M2-like TAMs in the TME, as indicated by the reduction of CD206^+^F4/80^+^ and CD163^+^ macrophage populations, without significantly affecting M1-like macrophages. This selective targeting led to a marked increase in the M1/M2 ratio, driven by the reduction of immunosuppressive M2 populations rather than an expansion of M1 subsets (**Figure 7**). Importantly, this modulation of the TME was accompanied by a significant enhancement of CD8^+^ T cell infiltration into the tumor (**Figure 8**). Both flow cytometry and immunohistochemical analyses revealed increased CD8^+^ T cell presence in MEL-dKLA-treated tumors, suggesting that depletion of M2 TAMs may relieve their suppressive effects on T cell trafficking and activation. These results are consistent with previous reports showing that M2 macrophages produce anti-inflammatory cytokines such as IL-10 and TGF-β, which inhibit T cell recruitment and effector function (38, 39). Furthermore, M2 macrophages can contribute to T cell exclusion by expressing immune checkpoint ligands and chemokines that create physical and chemical barriers within the TME (40). Notably, the combination of MEL-dKLA with immune checkpoint blockade has been reported to suppress breast cancer growth and metastasis (19). In line with these findings, the increased CD8^+^ T cell infiltration observed in our study suggests that MEL-dKLA may also synergize with immune checkpoint inhibitors, including anti-PD-1 or anti-CTLA-4 antibodies, in prostate cancer, thereby broadening its therapeutic potential. By targeting and eliminating M2-like TAMs, MEL-dKLA may dismantle this suppressive network, thereby enhancing CD8^+^ T cell access to tumor cells and strengthening anti-tumor immunity. Although MEL-dKLA primarily exerts macrophage-depleting activity, a fraction of macrophages may persist after treatment and undergo functional reprogramming. Metabolic regulation could also contribute to this process, particularly through the reciprocal utilization of L-arginine by ARG1 and iNOS, which represents a key axis in macrophage polarization. Further studies will therefore be required to determine whether MEL-dKLA alters ARG1 activity and/or NO production in these residual macrophages, thereby supporting their reprogramming toward an M1-like state.

Nevertheless, this study has several limitations. First, we evaluated the antitumor efficacy of MEL-dKLA mainly in the relatively immunogenic TRAMP-C2 prostate cancer mouse model. To enhance translational relevance, it will be important to assess its efficacy in castration-resistant prostate cancer (CRPC) models, which possess a more immunosuppressive tumor microenvironment. Recent studies have highlighted the therapeutic potential of converting “cold” tumors into “hot” tumors by reprogramming the immune milieu (41). In line with this concept, our future work will investigate whether MEL-dKLA–mediated depletion of M2-like TAMs can remodel the immunosuppressive CRPC microenvironment into a more immunogenic one, thereby providing a novel therapeutic strategy for advanced prostate cancer. Second, the current study primarily focused on a single dose of MEL-dKLA. Although we previously evaluated the dose-dependent antitumor activity of the melittin and selected the current dose based on those findings, directly predicting the maximum tolerated dose (MTD) of MEL-dKLA remains uncertain (17). Future studies testing multiple dose levels of MEL-dKLA will be necessary to define its MTD, which will serve as a critical translational index for clinical development.

In conclusion, this study demonstrates that MEL-dKLA selectively targets M2-like TAMs differentiated by tumor-conditioned medium derived from prostate cancer cells. MEL-dKLA induces apoptosis in these immunosuppressive macrophages, thereby inhibiting M2-like TAM-induced proliferation and invasion of prostate cancer. Furthermore, depletion of M2-like TAMs by MEL-dKLA in the TME significantly increases CD8^+^ T cell infiltration, suggesting that it reverses immunosuppression and restores antitumor immunity. These findings suggest that MEL-dKLA is a promising therapeutic candidate for prostate cancer.

## Data Availability

The original contributions presented in the study are included in the article/**Supplementary Material**, further inquiries can be directed to the corresponding author/s.
