# Supplementary figures and images for: Immunomodulatory peptide–drug conjugate MEL-dKLA suppresses progression of prostate cancer by eliminating M2-like tumor-associated macrophages
Source: Front Immunol. 2025 Sep 12;16:1652166. doi: 10.3389/fimmu.2025.1652166 (PMC12463987; doi:10.3389/fimmu.2025.1652166)

Supplementary Table 1. qRT-PCR primers for analysis


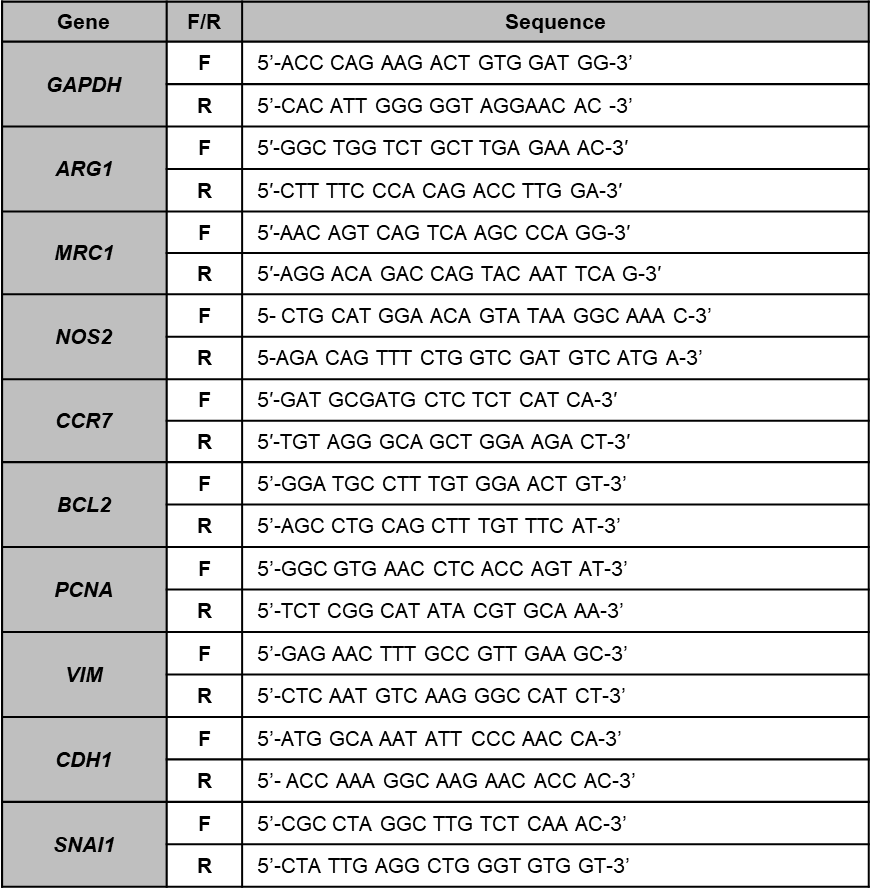

Supplement: Supplementary file 1 [file Table1.docx]
