# Supplementary material for: Immunomodulatory peptide–drug conjugate MEL-dKLA suppresses progression of prostate cancer by eliminating M2-like tumor-associated macrophages
Source: Front Immunol. 2025 Sep 12;16:1652166. doi: 10.3389/fimmu.2025.1652166 (PMC12463987; doi:10.3389/fimmu.2025.1652166)
Supplement: Supplementary file 2 [file Table2.docx]

Supplementary Material

# Supplementary Data


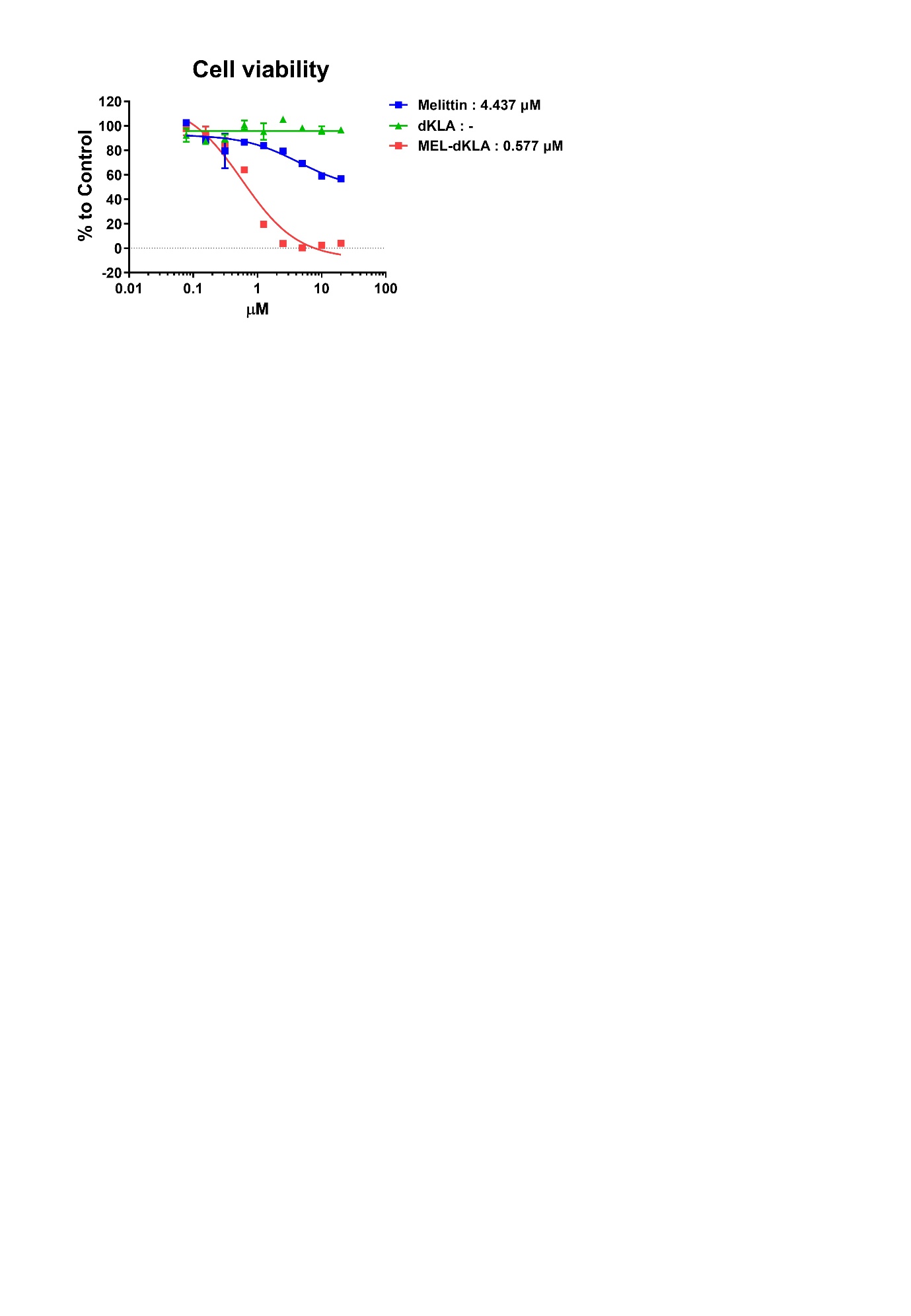


Supplementary Figure 1. Cytotoxicity of melittin, dKLA, and MEL-dKLA against M2-polarized macrophages. THP-1-derived M2 macrophages were treated with increasing concentrations of melittin, dKLA, or MEL-dKLA for 24 hours. Cell viability was assessed using the CCK-8 assay. Dose–response curves were generated, and IC₅₀ values were calculated using non-linear regression analysis. MEL-dKLA exhibited potent cytotoxicity with an IC₅₀ of 0.577 μM, whereas melittin showed moderate activity (IC₅₀ = 4.437 μM), and dKLA alone did not induce cytotoxicity within the tested concentration range. Data are presented as mean ± SD of three independent experiments.


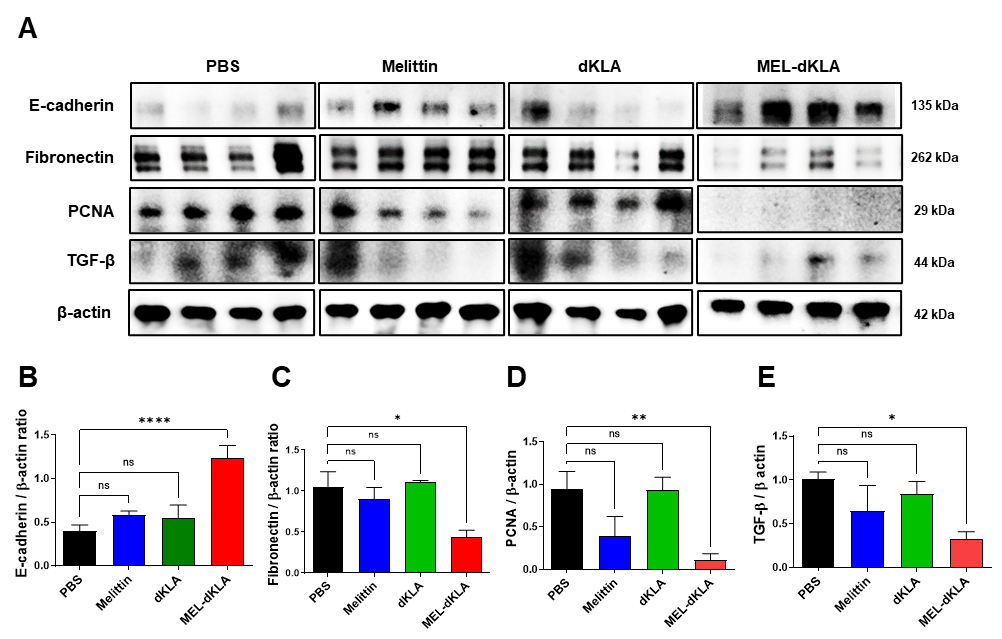


Supplementary Figure 2. Suppression of EMT and proliferative markers in tumor tissues by MEL-dKLA. (A) Representative Western blot images of E-cadherin, fibronectin, PCNA, and TGF-β in tumor tissues from mice treated with PBS, melittin, dKLA, or MEL-dKLA. β-actin was used as a loading control. (B–E) Densitometric quantification of protein bands normalized to β-actin: (B) E-cadherin, (C) fibronectin, (D) PCNA, and (E) TGF-β. Data are presented as mean ± SD (n = 4 per group). One-way ANOVA followed by Tukey’s post hoc test was used for statistical analysis. **p* < 0.05; ***p* < 0.01; *****p* < 0.0001; ns, not significant.
